# *Rickettsia parkeri* in Uruguay

**DOI:** 10.3201/eid1211.060577

**Published:** 2006-11

**Authors:** Richard C. Pacheco, José M. Venzal, Leonardo J. Richtzenhain, Marcelo B. Labruna

**Affiliations:** * University of São Paulo, São Paulo, SP, Brazil;; † University of La República, Montevideo, Uruguay

**Keywords:** *Rickettsia parkeri*, *Amblyomma triste*, isolation, Uruguay, letter

To the Editor: During 1990 in Uruguay, a rickettsiosis in the spotted fever group was presumptively diagnosed for 3 patients who had fever, an initial small maculopapulous lesion at the site of a tick bite on the scalp, and subsequent regional lymphadenopathy. Microimmunofluorescent serologic assay, with Rickettsia conorii as the sole antigen source, gave positive results for all patients, and these infections were presumptively identified as spotted fever caused by R. conorii (*1*). During 1993–1994, a total of 23 patients who had a history of tick bite, some of whom had exanthema and inoculation eschars, were identified from Canelones County, Uruguay. These patients had antibodies against R. conorii, according to microimmunofluorescence testing; however, R. conorii was again the sole antigen source used in the assay (*2*). Because 1 of the major limitations of serologic testing for diagnosis of rickettsioses is the cross-reactivity between different Rickettsia species, the association of R. conorii with the spotted fever group cases in Uruguay was considered inappropriate (*3*). In addition, R. conorii has never been found in the Western Hemisphere (*3*).

Amblyomma triste, a neotropical tick species with a variety of hosts, is the main tick species that feeds on humans in Uruguay and the primary candidate vector for tickborne rickettsioses in that country (*4*). A recent investigation demonstrated DNA of R. parkeri in A. triste ticks collected from humans and animals, indicating that this rickettsia could be the pathogenic agent of spotted fever group rickettsioses in Uruguay (*5*). In the United States, where A. maculatum ticks infected with R. parkeri have been reported since the 1930s, the role of this rickettsial agent as a human pathogen was confirmed only recently (*3*). Our study is the first to isolate R. parkeri from A. triste collected in Uruguay and confirms the presence of this emerging pathogen in South America.

During September 2004, 78 adult flat ticks (25 males, 53 females) identified as A. triste were collected from vegetation in the suburban area of Toledo Chico (34°44´53´´S, 56°06´19´´´W) in Canelones County, southern Uruguay. At the laboratory, the legs of live ticks were extirpated for DNA extraction, and the tick bodies were immediately frozen at -80°C. Each group of legs from 1 tick was subjected to DNA extraction by boiling at 100°C for 20 min as described (*6*). DNA extracted from each tick was tested by PCR by using primers CS-78 and CS-323 (Table), which targeted a 401-bp fragment of the citrate synthase gene (gltA) of possibly all Rickettsia species (*7*). For 2 ticks (1 male, 1 female) that had positive results with PCR testing, Rickettsia isolation in cell culture was attempted by using the shell vial technique with the following modifications: Vero cells inoculated with tick body homogenate were incubated at 28°C; the level of infection of cells was monitored by Gimenez staining of scraped cells from the inoculated monolayer; and a rickettsial isolate was considered established after 3 passages, each reaching >90% of infected cells (*7*).

**Table:** Primer pairs used for amplification of rickettsial genes

Primer pairs	Genes and primers	Primer sequences (5´→3´)	Fragment size (nucleotides)	Reference
	gltA			
1	CS-78	GCAAGTATCGGTGAGGATGTAAT	401	(7)
	CS-323	GCTTCCTTAAAATTCAATAAATCAGGAT		(7)
2	CS-239	GCTCTTCTCATCCTATGGCTATTAT	834	(7)
	CS-1069	CAGGGTCTTCGTGCATTTCTT		(7)
	ompB			
3	120-M59	CCGCAGGGTTGGTAACTGC	862	(8)
	120–807	CCTTTTAGATTACCGCCTAA		(8)
	ompA			
4	Rr190.70p	ATGGCGAATATTTCTCCAAAA	530	(9)
	Rr190.602n	AGTGCAGCATTCGCTCCCCCT		(9)

Rickettsiae were successfully isolated and established in Vero cell culture from the female tick. This isolate, designated as At5URG, has been deposited as a reference strain in the Rickettsial Collection of Faculty of Veterinary Medicine in the University of São Paulo. DNA extracted from infected cells of the third passage was tested by a battery of PCRs that used all primer pairs listed in the Table and targeted fragments of 3 rickettsial genes: gltA, ompB, and ompA. PCR products of expected size were obtained in all reactions and subjected to DNA sequencing as described (*6*). Fragments of 1,084, 775, and 491 nt of the gltA, ompB, and ompA genes, respectively, were obtained and showed 100% identity to the corresponding sequences available in GenBank (accession nos. U59732, AF123717, and U43802, respectively) for the Maculatum strain of R. parkeri from United States. Although isolation of Rickettsia from the male tick was unsuccessful, DNA extracted from remnants of the male and female ticks was tested by PCR (ompA, Table) and yielded product that after sequencing (491 nt) showed 100% identity to the R. parkeri sequence from GenBank (U43802).

These procedures enabled the identification of R. parkeri in 2.56% of the A. triste ticks from Uruguay. Previous findings of R. parkeri DNA in A. triste ticks from Uruguay (5) are corroborated by our isolation of a Uruguayan strain of R. parkeri in cell culture. The only other country where R. parkeri has been previously reported is the United States, where it is associated with A. maculatum ticks and is the causative agent of an emerging rickettsiosis (*3*). As A. maculatum and A. triste are established in at least 12 other Latin American countries (*10*), the distribution of R. parkeri in the Americas is likely continental. Finally, our results corroborate recent reports (*3**,**5*) that suggest R. parkeri is the causative agent of previously reported cases of rickettsiosis in Uruguay.
